# The type IV pilus steering committee: how Pil-Chp controls directional motility

**DOI:** 10.1128/jb.00396-24

**Published:** 2025-09-19

**Authors:** Kaitlin D. Yarrington, Dominique H. Limoli

**Affiliations:** 1 Department of Microbiology and Immunology, University of Iowa 4083, Iowa City, Iowa, USA; 2 Department of Bioengineering, Phil and Penny Knight Campus for Accelerating Scientific Impact, University of Oregon, Eugene, Oregon, USA; 3 Institute of Molecular Biology, University of Oregon, Eugene, Oregon, USA; 4 Department of Biology, Indiana University 1772, Bloomington, Indiana, USA; University of California San Francisco, San Francisco, California, USA

**Keywords:** type IV pili, Pil-Chp, chemotaxis, *Pseudomonas aeruginosa*

## Abstract

Many microbial species live on surfaces and employ various strategies for initiation of and survival within a surface-attached community. One such strategy implemented by many bacterial species is to move across surfaces using grappling hook-like appendages called type IV pili (TFP) which extend, attach to the surface, and retract to pull the cell body forward. In the bacterium *Pseudomonas aeruginosa*, TFP motility, or twitching, is controlled by the Pil-Chp system. *P. aeruginosa* uses this system to traverse surfaces and gather information about the local chemical and physical environment. The Pil-Chp system shares many similarities to the well-studied flagellar chemotaxis system (Che), which biases locomotion of swimming cells up or down gradients of chemical stimuli. However, many important differences have been described, while others await discovery. Some of these differences have even led to speculation that chemotaxis may not be a primary role for Pil-Chp. Thus, recent studies have focused on addressing whether *P. aeruginosa* uses chemotaxis to bias the direction of motility on a surface, and if so, what role does Pil-Chp play in this process? In this review, we focus on current progress in the field toward gaining insight into these questions.

##  INTRODUCTION TO CHEMOTAXIS

Microbes are constantly receiving and interpreting inputs from complex extracellular environments. These signals harbor information about the state of their surroundings, including nutrient availability, the metabolite profile, and secreted products from other species that may indicate whether the neighboring microbes are beneficial or threatening. By sensing each type of cue, microbial species are poised to respond to the ever-changing components of their environment for the best chance of survival. Microbes often employ a myriad of receptors to sense diverse signals, which relay information through signaling cascades for a variety of outputs, such as differential regulation of specific nutrient acquisition pathways or engaging motility appendages to maneuver surroundings.

Organismal response to environmental stimuli through locomotion can occur in multiple ways. For example, an organism may move in random directions (referred to as kinesis) but change the speed of movement (orthokinesis) or the rate of turning (klinokinesis). Alternatively, organisms can respond with a directional response to stimuli (taxis). Each is further defined by the type of stimulus, e.g., chemokinesis, chemotaxis, phototaxis, etc. (1). Organisms that locomote on surfaces also specifically experience mechanical forces due to cell-to-substratum interactions and hydrodynamic flow, referred to as mechano- or surface sensing. Thus, bacteria traveling on surfaces are likely to simultaneously experience mechanical and chemical cues; how these are sensed and translated into kinesis and/or taxis is an active field of investigation.

Directional motility by bacteria in response to a chemical signal gradient, or chemotaxis, canonically begins with a diffusible ligand binding a methyl-accepting chemotaxis protein (MCP) receptor. In response, the MCP controls a downstream signaling cascade leading to directional motility (2). Two common bacterial motility appendages include flagella, which mediate swimming motility through liquid environments, and type IV pili (TFP), which control twitching motility on surfaces. Together, flagella and TFP also coordinate swarming motility on surfaces. Early studies on bacterial chemotaxis dissected flagella-mediated swimming chemotaxis pathways and chemoeffectors in the Che system of *Escherichia coli*. However, more recent studies have also revealed that bacterial species can perform chemotaxis on surfaces (3, 4).

**Fig 1:**
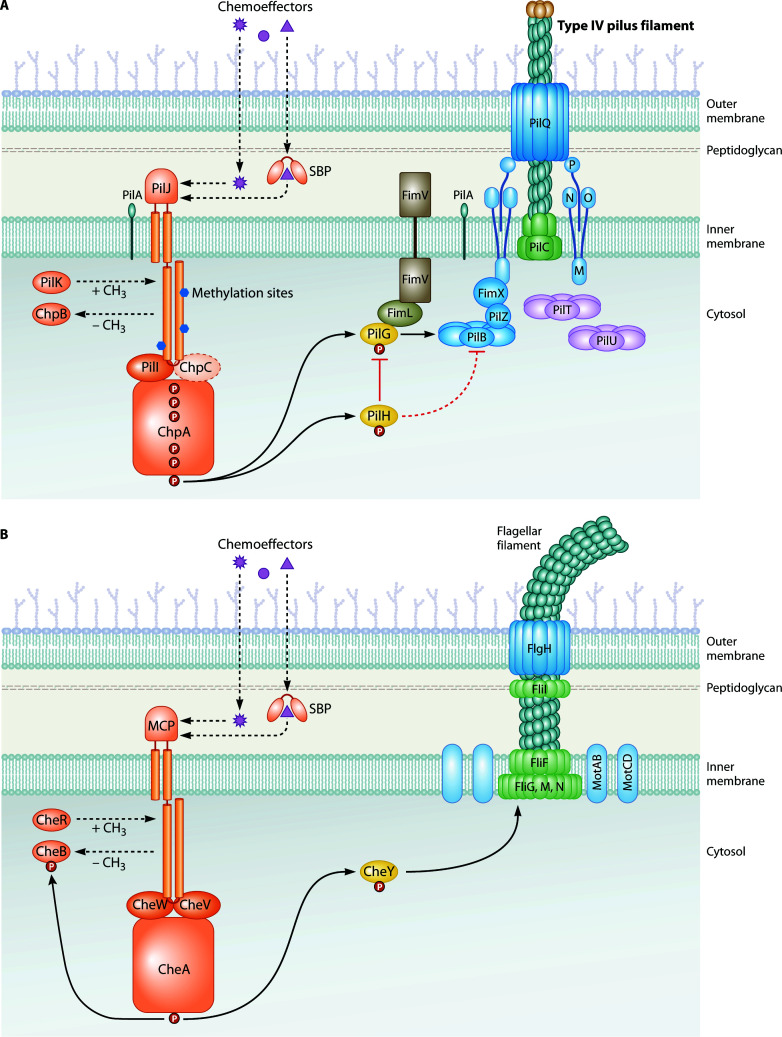
The Pil-Chp system controls TFP-mediated chemotaxis, and the Che system controls flagella-mediated chemotaxis in *P. aeruginosa*. (**A**) Schematic of Pil-Chp and the basic components of the TFP machinery. At the inner membrane platform protein, PilC (trimeric protein in green) (6), PilA monomers (teal) are polymerized to make a growing TFP filament that is extended out of the cell body through the outer membrane porin, PilQ (protein in light blue). At the tip of the pilus sit the minor pilins and PilY1 adhesive tip protein (tan). PilMNOP serves as the alignment complex of the inner and outer membrane machinery components (proteins in light blue, labeled by last letter only). The inner membrane-bound, MCP, PilJ (orange), binds chemoeffectors (dark purple) in the periplasmic ligand-binding domain and transduces the signals through CheW-like linker proteins, PilI and ChpC, in the cytoplasm to activate the histidine kinase, ChpA (proteins in orange). PilI is essential for twitching motility, whereas ChpC is not and instead may have a role in signal transduction of chemosensory signals (7–9). Phosphotransfer through the Xpt domains of ChpA (represented by dark orange circles labeled with “P”) leads to activation of the response regulators PilG and PilH (proteins in yellow) for control over the localization and activity of the pilus extension protein complex, PilB-FimX-PilZ, an extension ATPase, degenerate c-di-GMP-binding diguanylate cyclase, and degenerate PilZ domain c-di-GMP effector protein, respectively (proteins in azure blue). PilG polar localization is dependent on hub proteins FimV and FimL (proteins in brown) and can be inhibited by PilH (10–12). PilH direct inhibition of PilG also indirectly inhibits PilB localization at the machinery complex (represented by dotted line), priming cells for reversals. The ChpB response regulator is not thought to be phosphorylated (13). ChpB, a methylesterase, and PilK, a methyltransferase, (proteins in orange) serve as the Pil-Chp chemotaxis adaptation proteins, which likely modify the sensitivity of PilJ to chemoeffector ligands through the removal or addition of methyl groups at the cytoplasmic methylation sites (blue hexagons), respectively. Pil-Chp also includes two retraction ATPases, PilT and PilU (proteins in light purple). PilU is dependent on PilT for activity (14); however, how their localization and depolymerization activity are coordinated by the rest of the Pil-Chp system is still not fully understood. (**B**) Schematic of the Che chemotaxis system and basic components of the flagellar machinery. The flagellar filament (teal) is polymerized and extended out of the cell body through the flagellar membrane complex that spans the entire *P. aeruginosa* membrane. The complex consists of several FliF proteins, which make the MS-ring that sits in the inner membrane (proteins in green). Attached to the MS-ring and extending into the cytoplasm is the C-ring consisting of several FliGMN motor switch proteins (proteins in green), which determine the direction of flagellar rotation. The P-ring in the peptidoglycan layer is composed of many FliI proteins (green), and the flagella filament extends out of the cell body through the outer membrane L-ring made of FlgH proteins (light blue). Rather than constant polymerization and depolymerization of the pilus filament, controlled by extension and retraction ATPases to generate movement, the flagellar filament is polymerized once, and rotational movements are powered by two complexes of paired stators, MotAB and MotCD (baby blue). In *P. aeruginosa,* 23 MCPs (represented by one orange MCP) are associated with the Che I system, and 1 MCP is associated with the Che II system. Similar to Pil-Chp, the Che MCPs sense chemoeffectors (dark purple) in the ligand-binding domains and transduce the signal through the CheW and CheW-like, CheV linker proteins to the histidine kinase CheA (proteins in orange). CheA only has one Hpt phosphorylation domain (dark orange circle labeled with “P”) and phosphorylates the response regulators CheY (protein in yellow) and CheB (protein in orange). Phosphorylated CheY interacts with the flagellar motor switch complex (FliGMN) to change the direction of flagellar rotation from counterclockwise to clockwise, resulting in a turn. In the Che system, CheB and CheR serve as the predicted methylesterase and methyltransferase chemotaxis adaptation proteins (orange), respectively, and are thought to operate similarly to the predicted functions of ChpB and PilK. While Che MCPs in other organisms do have methylation sites, the number and location of these sites within the MCP cytoplasmic domains can vary, and no methylation sites have been explicitly predicted in *P. aeruginosa* Che MCPs; therefore, no methylation sites are shown on the schematic. For PilJ and Che MCPs, there may be chemoeffector ligands that can bind directly to the ligand-binding domain and other ligands that may bind indirectly, such as through the help of a solute-binding protein (SBP; protein in orange holding a chemoeffector).

The environmental bacterium and opportunistic pathogen *Pseudomonas aeruginosa* has both polar TFP and a single, polar flagellum. Therefore, this species serves as an excellent model to investigate flagella and pili-associated chemotaxis pathways within the same organism (Fig. 1). Both types of motilities have been extensively studied, but far more is known about how flagella-mediated motility operates and how the associated Che I and II systems modulate flagellar chemotaxis in *P. aeruginosa*. While both Che I and II are homologous to much of the well-studied *E. coli* Che flagellar chemotaxis system, Che I is predicted to respond to chemoeffector signal input from 23 of the 26 MCPs in *P. aeruginosa*, while Che II is only associated with one chemoreceptor for flagella-driven aerotaxis (5). Meanwhile, each of the remaining two predicted chemoreceptors, PilJ and WspA, is associated with TFP motility and the Wsp surface sensing system, respectively (5).

Transformative studies tracking swimming bacterial motion in isotropic solutions and gradients by Howard Berg in the 1970s revealed that *E. coli* swims via smooth runs that are interspersed randomly by tumbles, resulting in a change in cell body orientation. The cell then resumes smooth runs and swims off in a new direction (15). Smooth runs are generated by flagellar rotation in the counterclockwise direction, which allows the peritrichous flagellar filaments of *E. coli* to work together in a bundle. A tumble is generated by a change in flagellar rotation to the clockwise (CW) direction, which results in the bundled filaments coming apart (16). Under chemical gradients, motion is biased up attractant or down repellent gradients by changing the frequency of runs and tumbles to maintain smooth runs or reorient the direction of cellular motion, respectively. Instead of the peritrichous flagellar organization of *E. coli, P. aeruginosa* possesses a single polar flagellum. While this organization generates a pause and turn upon switching to CW rotation, instead of a tumble, flagellar chemotaxis follows largely the same principles as described for *E. coli* (15, 17).

**Fig 2:**
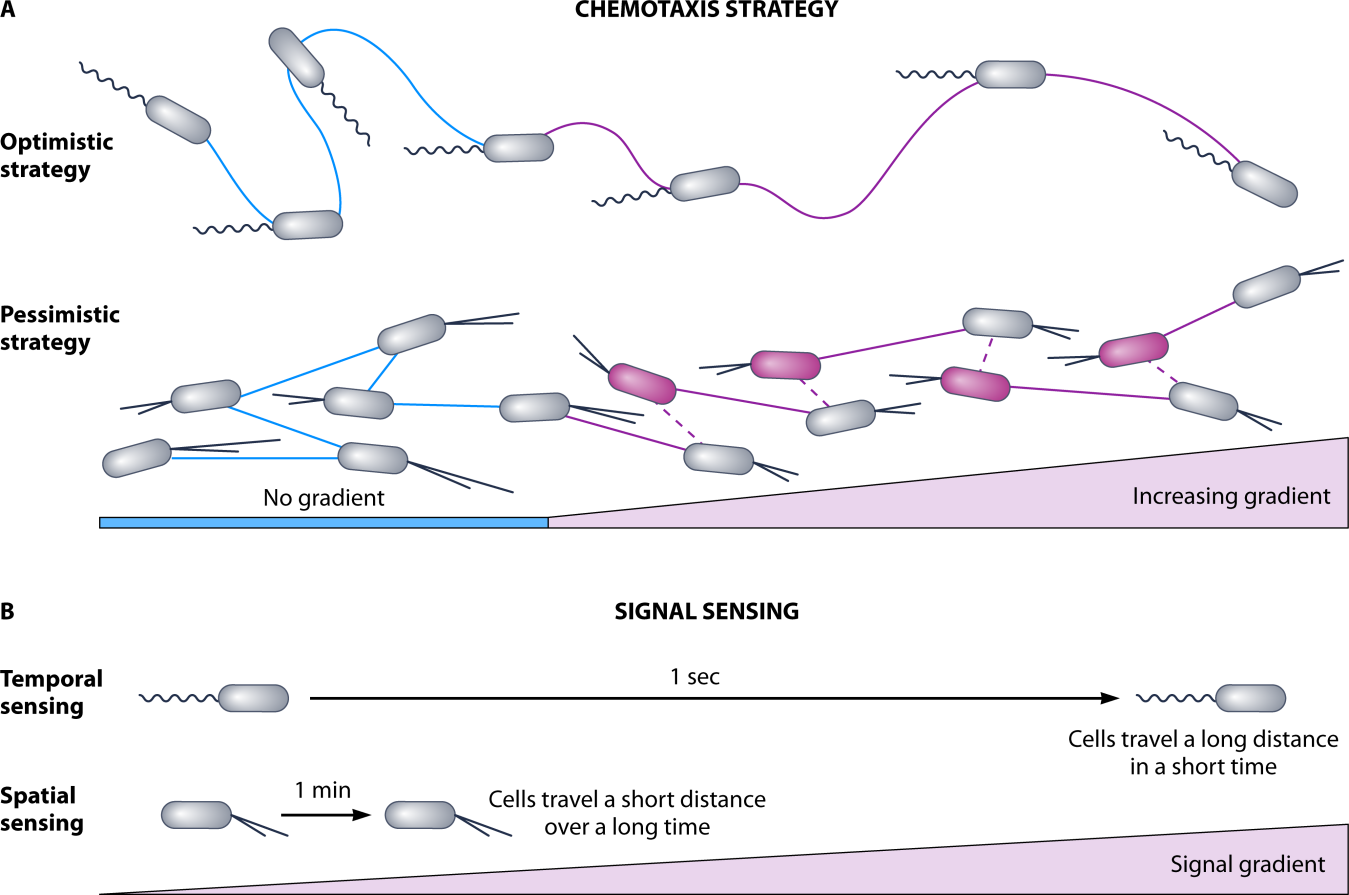
Key differences in flagella- versus pilus-mediated motility and chemotaxis movements. (**A**) A single, polar flagellum propels *P. aeruginosa* through liquid in a “run-reverse-turn” pattern, allowing cells to randomly move in all directions. When cells undergo a “reverse and turn” movement (curvier blue lines), the cell body orientation changes from the previous position, leading to a new trajectory direction on the next run (straighter blue lines). In the absence of a signal gradient, swimming cells navigate the environment with continual repetition of “run-reverse-turn” movements with more frequent changes in direction. However, in the presence of a chemoattractant gradient, swimming cells navigate the gradient with an “optimistic” strategy in that when they sense they are moving toward an increase in the signal concentration (purple cells), cells decrease the frequency of reverse and turn events and increase flagella-mediated runs (purple lines) up the increasing gradient. On a surface, *P. aeruginosa* uses TFP that extend and retract from the poles to twitch back and forth across surfaces (blue lines). Changes in motility direction occur through reversals in which higher TFP extension and retraction activity at the previous leading pole switches to the opposite pole, which becomes the new leading pole. In the presence of a chemoattractant gradient, *P. aeruginosa* twitching cells employ a “pessimistic” strategy in which cells (gray cells) take steps up the gradient after a “correct” reversal (longer, purple solid line). However, when cells make an “incorrect” reversal (shorter, purple dashed lines) and sense they are moving toward a decreasing chemoattractant gradient (purple cells), shorter steps are taken, and there is an increase in reversal events that occur to extend pili from the other pole and move up the gradient again. (**B**) During flagella-mediated chemotaxis, cells temporally sense changes in the signal gradient. The fast movements generated by the flagellum enable cells to sample more of the gradient within a time frame and thus larger differences in signal concentration. In contrast, spatial sensing is proposed to be used in TFP-mediated chemotaxis as the slow-crawling cells sample less of the gradient in the same amount of time. Under this model, twitching cells are able to sense a chemical gradient in the absence of changes in movement or time.

Flagellar response to a chemical gradient begins with a chemoeffector binding a methyl-accepting chemoreceptor (MCP; Fig. 1B). The signal is then transmitted to the histidine kinase, CheA, through a linker protein, CheW. If the signal is a repellent, CheA then phosphorylates the response regulator CheY, which interacts with the flagellar motor to change the direction of flagellar rotation to CW and generate a tumble/turn (Fig. 1B). If the signal is an attractant, then CheA activity is reduced and thus also the frequency of CheY-mediated tumbles/turns. To detect a concentration gradient, the sensitivity of the MCP is regulated by two opposing enzymes, a methyltransferase (CheR) and a methylesterase (CheB), which add and remove methyl groups from the MCP, respectively (Fig. 1B). CheB activity is enhanced upon phosphorylation by CheA. Thus, following a tumble/turn, the MCP is demethylated, which reduces its sensitivity to the ligand so that only a higher concentration can activate the system again. Thus, bacterial chemotaxis systems respond not to an absolute concentration of solute, but to a change in concentration. However, since bacterial cells are very small and most chemoeffectors are highly diffusible, bacterial cells are canonically thought to be incapable of sensing a concentration gradient across the cell body. Thus, cells must “outswim” the diffusion constant of the solute to determine if the concentration is changing (18). This results in swimming cells integrating detection of concentration over time to determine if the concentration has changed from what it was a moment ago, a feature provided by the methyl modification enzymes (Fig. 2).

Whether twitching cells follow a similar strategy to move up chemoattractant gradients is not fully understood. Compared to planktonic swimming cells sampling gentle chemoattractant gradients by traveling a full body length or more between tumbling events, surface-associated twitching motility is hundreds to thousands of times slower, and steep, varying gradients characterize the chemotactic landscape (Fig. 2). Therefore, it is highly plausible that the TFP-mediated chemotaxis strategy deviates from the flagella-mediated mechanism (Fig. 2), which will be discussed in detail in the following sections.

## OVERVIEW OF THE PIL-CHP SYSTEM

TFP-mediated motility, or twitching motility, occurs through the grappling hook activity of the pilus, which undergoes episodes of extension, substrate attachment, and retraction which pulls the cell body along the surface (19). Each TFP filament is composed of many PilA monomers (Fig. 1A). TFP dynamics are coordinated by the single extension ATPase, PilB, and two retraction ATPases, PilT and PilU, to respectively polymerize PilA monomers into the growing pilus filament and depolymerize the filament back into PilA monomers within the cell membrane (Fig. 1A) (19–21). The direction of twitching motility is thought to be controlled by preferential extension of pili at the pole facing the direction of movement, referred to as the leading pole. Cells are predicted to change direction by extending pili from the opposite pole, reversing the direction of cellular movement along the long axis of the cell body and swapping leading poles (10).

Proteins encoded in the putative pilus chemosensory pathway, Pil-Chp, share sequence and structural homology to those in the flagellar chemotaxis pathway, Che. Based on such homology, the pilus-associated MCP, PilJ, is predicted to bind chemical solutes and transmit the signal through the histidine kinase ChpA to the CheY-like response regulators, PilG and PilH, which are thought to modulate pilus polarity to produce a cellular reversal (Fig. 1A). The Pil-Chp-associated methyltransferase and methylesterase (PilK and ChpB, respectively) are predicted to add and remove methyl groups to PilJ to modulate receptor sensitivity to ligands and allow the system to adapt to changing ligand concentrations (Fig. 1A) (10, 22, 23). While many of these predicted functions have been experimentally validated, several notable differences from the flagellar system have been discovered, detailed in the following sections.

### The methyl-accepting chemoreceptor, PilJ

One primary difference between the flagellar and pilus chemosensory systems is the number of chemoreceptors associated with each pathway. Of the 26 predicted MCPs in *P. aeruginosa*, 24 of them are assigned to the flagellar-associated chemotaxis pathways and bind to a range of solutes, such as amino acids and sugars (5). Meanwhile, the only receptor predicted to associate with Pil-Chp is PilJ. Additionally, deletion of *pilJ* renders cells unable to twitch (24, 25), while deletion of Che-associated MCPs leads to defective directional movement of swimming cells, but leaves baseline motility intact (26). The necessity of PilJ in twitching motility is derived in part through its role in activation of PilG, which activates the main adenylate cyclase, CyaB, to elevate production of the second messenger cyclic adenylate monophosphate (cAMP). cAMP then binds to and activates the virulence factor transcriptional regulator, Vfr, which subsequently increases expression of many genes including upregulation of *pil-chp* and TFP machinery genes (27). PilJ also has a well-described role in surface/mechanosensing (please see the following expert reviews for more details, [28–31]). To dissect the possible chemosensory role of PilJ from its role in motility regulation, recent studies have interrogated the structure of the PilJ ligand-binding domain (LBD), performed targeted mutagenesis of the chemosensory regions of PilJ, and sought to identify ligands that bind to PilJ.

**Fig 3:**
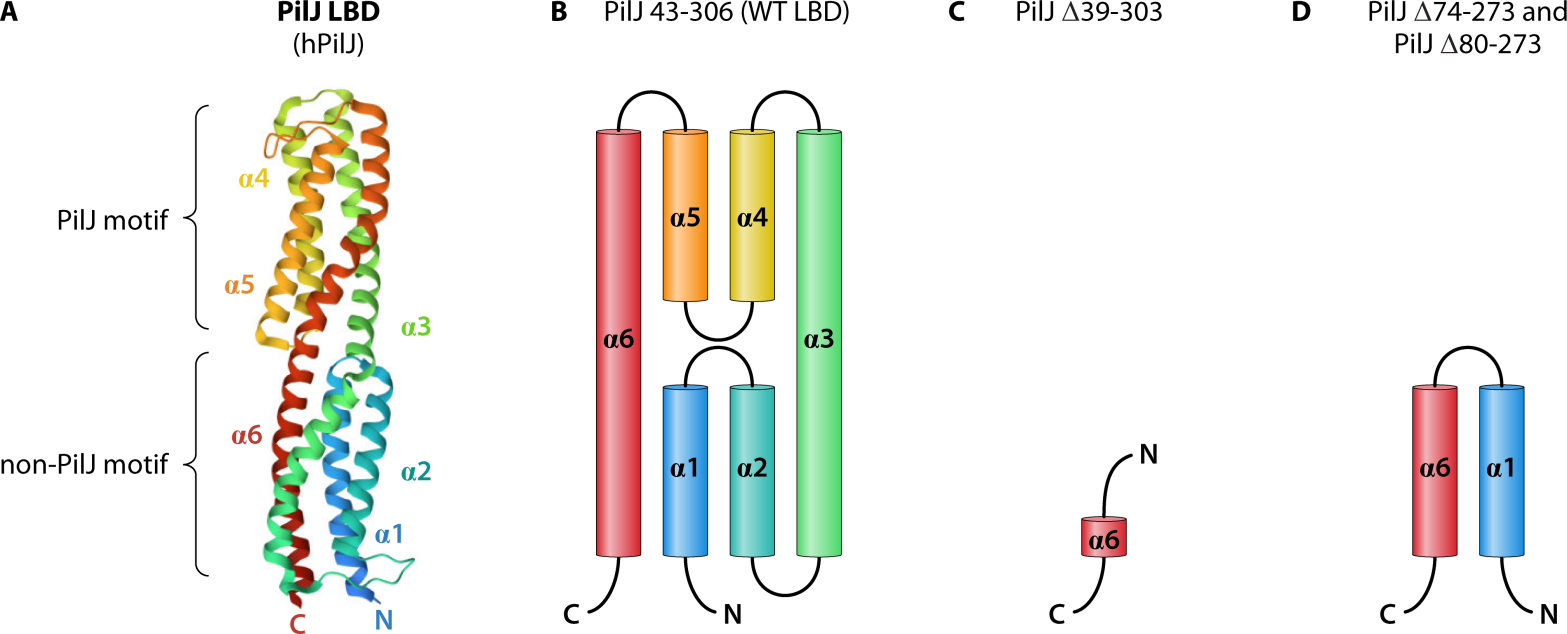
Crystal structure of the PilJ LBD (PDB ID: 8WDF)**.** (**A**) The LBD of *P. aeruginosa* chemoreceptor PilJ folds into an HBM-type LBD rather than a pilJ-type motif. Yet, the PilJ LBD still contains a PilJ motif, canonically in pilJ-type LBDs. Therefore, Cui et al. proposed sub-classifications for LBDs with PilJ motifs and placed the *P. aeruginosa* PilJ LBD in the hybrid PilJ (hPilJ) sub-classification as it contains both a PilJ motif and a non-PilJ motif (33). (**B**) The PilJ LBD structure in (**A**) generated from PilJ amino acids 43–406 consists of four shorter alpha helices and two longer alpha helices. (**C**) PilJ LBD mutants lacking amino acids 39–303 only retain a tiny fragment of alpha helix 6 and cannot chemotax, yet remain fully motile. (**D**) In contrast, PilJ LBD mutants lacking amino acids 74–273 or similarly lacking amino acids 80–273 retain all of alpha helix 1 and half of alpha helix 6. However, these mutants are nearly non-motile.

*PilJ ligand-binding domain.* Most *P. aeruginosa* membrane-bound MCPs contain a single LBD, but based on amino acid sequence similarity, PilJ was initially predicted to possess two LBDs, each with an independent ligand-binding pocket (5, 32). While there are many classes of LBDs, both predicted LBDs in PilJ had long been classified as “pilJ-type” LBDs (5, 7, 32). However, the recently solved crystal structure of the Pil-Chp PilJ LBD revealed more structural similarity with a single helical bimodular motif (HBM)-type LBD, like the chemoreceptor McpS in *Pseudomonas putida* and the Tar receptor in *E. coli*, rather than the two pilJ-type domains initially predicted (Fig. 3A and B) (33). This structure provides useful information to further probe the role of PilJ as a chemoreceptor. Yet, even with atomic structural data, there is still no evidence for the site of ligand binding or what ligands may bind. As an additional unique feature to PilJ, Cui et al. provide evidence that PilJ exhibits trimeric oligomerization (33). Most of the described Che-associated Mcps form dimers that are assembled into “trimers of dimers” (34, 35). The difference in oligomerization may further imply differences in how PilJ relays signals through the pathway. Interestingly, other HBM-type LBDs often possess two distinct ligand-binding sites, suggesting this class of MCPs may sense a broader range of signals and be able to sense multiple signals simultaneously (36, 37). Ligands that activate the PilJ periplasmic domain could bind either directly to PilJ or indirectly via a solute-binding protein (SBP), of which there are 96 predicted SBPs in *P. aeruginosa* (Fig. 1) (38). While fewer examples of this mode of binding have been shown thus far, the *P. aeruginosa* MCP, CtpL, is likely activated in this way (39). Specifically, inorganic phosphate binds the SBP PstS (part of the phosphate-specific transport system), which has high affinity for binding to CtpL (39).

*PilJ methylation.* In addition to the LBD, MCPs contain specific glutamate and glutamine residues that undergo methylation and demethylation, generally associated with chemosensory adaptation, a process that fine-tunes the sensitivity of the MCP to gradient signals. PilJ contains at least three likely methylation modification glutamine and/or glutamate sites within its cytoplasmic region, based on the conserved methylation motif, [A/S/T/G]-[A/S/T/G]-X-X-[E/Q]-[E/Q]-X-X-[A/S/T/G]-[A/S/T/G] (Fig. 1A) (8, 9, 40, 41). The roles of each methylation site are still unclear, but recent work suggests that the modification of each site influences PilJ function in unique ways (42). At least one methylation site is necessary to respond to chemoattractant stimuli, while the other two are important for functional twitching and may have additional roles in fine-tuning PilJ signal sensitivity to chemoeffectors. While methylation of these specific sites has yet to be shown, the methyl modification enzymes encoded within the Pil-Chp system, a methyltransferase, PilK, and methylesterase, ChpB, are necessary for PilJ methylation *in vivo* (23) and for TFP directional motility (Fig. 1A) (42).

*PilJ interactions with linker proteins.* Canonically, upon ligand binding to an MCP, the signal is transmitted to the chemosensory system histidine via a CheW linker protein (Fig. 1B). Within the cytoplasmic tip of PilJ, conserved sequences facilitate interactions with the kinase and CheW-like linker protein interactions (9). Two CheW-like linker proteins, PilI and ChpC, are associated with Pil-Chp, in contrast to the typical single linker protein in the *P. aeruginosa* and *E. coli* Che chemotaxis systems (Fig. 1A). While PilI is essential for twitching motility and production of cAMP, ChpC is largely dispensable for these functions (Fig. 1A) (43, 44). However, ChpC may be important for modulating twitching response to a variety of signals (38), hinting at potential roles for Pil-Chp to engage a primary linker protein designated solely for twitching motility signaling and an accessory linker when transmitting sensory information of chemotactic signals. With clues to several behaviors occurring through Pil-Chp, it is not unreasonable that multiple linkers would be involved to distinguish signals and tightly regulate kinase activity to correctly control each output. Additionally, whether PilI is always part of the core signaling complex and ChpC joins for enhanced signaling transmission to certain cues, or if only one linker engages with the core signaling complex at a time, has yet to be determined.

### The histidine kinase, ChpA

The Pil-Chp histidine kinase, ChpA, adds another layer of complexity to this already unique system. Unlike the simple, single histidine phosphotransfer (Hpt) domain found in the flagellar kinase CheA, ChpA is an unorthodox CheA-like kinase at over three times the size, possessing a combination of at least nine histidine, threonine, and serine phosphotransfer (“Xpt”) domains (Fig. 1) (13, 45). Additionally, ChpA contains an *in cis* CheY-like receiver domain that is essential for twitching motility, which is more similar to the domain organization of the *M. xanthus* TFP chemotaxis kinase, FrzE, than to CheA in *P. aeruginosa* (46). Why ChpA contains so many Xpt domains is poorly understood, but one hypothesis is that additional domains broaden the phosphorylation repertoire to control the activity of the two response regulators in the system. While much has been learned about the hierarchy and direction of phosphoryl flow among the many ChpA Xpt domains, there is more to be discovered about how phosphotransfer activity of each of the nine Xpt domains is influenced by various inputs into PilJ and whether the order or number of activated Xpt domains depends on cues transmitted through PilI versus ChpC. Distinct phosphotransfer regimes may be necessary for PilJ/ChpA to modulate chemo- and mechanosensing simultaneously. In addition, only five Hpt domains of the nine total ChpA Xpt domains possess phosphotransfer ability, at least, in the absence of known signals (13, 45). The roles of the four non-phosphorylated Xpt domains in enhancing ChpA phosphorylation activity, whether modulating the activity of the five Hpt domains that undergo phosphorylation or possibly exhibiting phosphotransfer activity that was previously obscured in the absence of signal transmission from a specific ligand, remain unknown. With half of the domains unable to experience phosphorylation, it is further possible that these ChpA Xpt domains could function as protein-binding scaffolds even if they cannot participate in the phosphotransfer reaction.

### The response regulators, PilG and PilH

Histidine kinases customarily phosphorylate the chemotaxis system response regulators, leading to their activation and subsequent control over motility machinery. In the *P. aeruginosa* Che system, the phosphorylated CheY response regulator controls the direction of motor rotation of the single, polar flagellum (Fig. 1B). However, there are multiple pili deployed from both poles in *P. aeruginosa,* and two response regulators, PilG and PilH, are predicted to control pilus activity via interactions with the three TFP ATPases (Fig. 1A) (21, 47). While deletion of *pilG* or *pilH* results in reduced motility in macroscopic subsurface twitching motility assays, Δ*pilG* cells have reduced surface pili and reduced motility at the single-cell level and Δ*pilH* cells have increased surface pili and increased single-cell motility (21, 42, 48, 49). The phosphoacceptor sites of PilG and H are necessary for these functions. Thus, the initial interpretation of these data was that upon phosphorylation by ChpA, PilG enhances the function of the extension ATPase PilB and PilH of the primary retraction ATPase PilT. Recent data lend further insight into this model, suggesting that PilG controls the localization of PilB at the poles (Fig. 1A). PilH was found to also target PilB, but indirectly through inhibition of PilG phosphorylation (Fig. 1A) (10, 22). A detailed discussion of the localization of Pil-Chp proteins and how they may direct motility will be outlined in the section Control of Directional Motility on a Surface.

## CHEMICAL SIGNALS DIRECTING TWITCHING MOTILITY

Early studies on PilJ identified phosphatidylethanolamine (PE) and its long-chain unsaturated fatty acid degradation products as chemoattractants, dependent upon the periplasmic region of PilJ, where the LBD is located (24, 25, 50). While molecules like PE possess surfactant activity that may physically contribute to altered motility, Kearns et al. demonstrated that PE primarily acts as a transduced chemical signal rather than a surfactant-mediated surface lubricant due to the specificity of the response for certain PE species (25). The authors concluded that PE-directed motility may result from a chemokinetic response, since a PE gradient was not required to increase the speed of twitching motility. However, *pilJ* mutants had severely diminished twitching motility (24, 25); therefore, fully decoupling twitching versus a chemotactic response in these mutants is difficult to assess.

Our coculture studies of *P. aeruginosa* with *Staphylococcus aureus* revealed a TFP-mediated directional response of *P. aeruginosa* toward *S. aureus* cells and cell-free supernatant. The primary signals identified thus far are a family of *Staphylococcal* secreted peptides called phenol-soluble modulins (PSMs) (51). PSMs are short, alpha helical amphipathic peptides with cytotoxic activity toward eukaryotic cells, but typically do not affect the viability of prokaryotic organisms (52, 53). As with PE, PSMs possess surfactant activity, yet directional motility is specific for certain PSMs (α1, α3, and δ), suggesting a transduced chemical signal, instead of a general response (54). Initial investigation of a role for PilJ suggested it is dispensable for directional motility toward *S. aureus* (51). This conclusion was based on live cell microscopy of a *pilJ* mutant. While this mutant was non-motile in macroscopic assays, it appeared to retain directional motility toward *S. aureus* at the single-cell level. Subsequent analyses revealed the residual motility of the *pilJ* mutant to be flagellar, instead of TFP mediated, since a double Δ*pilJ* Δ*flgK* (flagellar hook associated protein) mutant was non-motile, even at the single-cell level in the presence of *S. aureus* (42). These studies emphasize the challenges in visually differentiating motility types and the importance of the genetic background of strains used to examine motility phenotypes. Thus, all our subsequent studies of genes necessary for TFP-mediated motility are performed in both wild-type and flagellar-deficient backgrounds (42).

In an attempt to generate a *pilJ* mutant which retains baseline twitching motility, we constructed several deletions in the ligand-binding domain of PilJ (42). We determined that when most of the predicted LBD region (residues 39–309) was deleted (Fig. 3C), chemotaxis toward PSMs was lost, while cells were still motile (33, 42). This contrasts with most other mutants that have been generated in the LBD region of PilJ. Namely, another group tested a PilJ mutant lacking residues 74–273 (55), and we similarly tested a mutant without residues 80–273 (Fig. 3D) and found that both have severe twitching defects to the point that chemotaxis could not be assessed (24, 42). Since the structure of the PilJ LBD by Cui et al. is now available (Fig. 3A and B), we may have more understanding of why each of these mutants displays certain phenotypes (28). The expression construct used to obtain the crystal structure consisted of PilJ residues 43–306 is a close match to the residues deleted in the LBD mutant that could twitch, but not chemotax (Fig. 3A through C) (33, 42). This suggests that the *pilJ*_ΔLBD1-2_ mutant (lacking residues 39–303) is a near-complete deletion of all six alpha helices that make up the entire LBD (Fig. 3C). Meanwhile, the PilJ mutant missing only residues 74–273 appears to still have the entire first alpha helix and half of the sixth alpha helix (Fig. 3D) (24, 33). One hypothesis may be that retention of a few helix fragments in the PilJ_Δ74-273_ protein is more destabilizing to the secondary structure of PilJ than complete loss of all helices; however, protein levels of PilJ_Δ74-273_ compared to wild-type PilJ have not been tested. Accordingly, it is still unknown how the secondary or tertiary structure of the cytoplasmic domains of PilJ changes in each of these LBD mutants and the impact of these potential changes on the downstream Pil-Chp cascade.

Additionally, cleverly controlled microfluidic gradients have also allowed for the discovery of likely chemoattractants that can stimulate *P. aeruginosa* twitching cells. Oliveira et al. showed that cells able to attach and move along the surface of a microfluidic chamber can bias motility toward increasing concentrations of succinate, dimethyl sulfoxide (DMSO), and, unexpectedly, antibiotics, all of which are dependent on Pil-Chp, particularly the response regulator PilG (3, 55). This group termed *P. aeruginosa*-biased movements toward lethal concentrations of antibiotics as “suicidal chemotaxis,” given that many cells cluster at the highest concentration of antibiotics and die (55). Of note, the *S. aureus* PSMs we identified as a *P. aeruginosa* chemoattractant have been previously reported to possess cytotoxic properties against eukaryotic host cells (42, 51, 53). While cytotoxic effects against *P. aeruginosa* were not observed, it is not unreasonable to propose that small peptides and antibiotics may similarly induce a stress-like TFP-mediated chemotaxis response (54). This begs the question of why *P. aeruginosa* would move toward harmful signals. In the antibiotic and PSM signal studies, *P. aeruginosa* was also noted to upregulate production of virulence factors while moving toward these gradients, relative to cells undergoing twitching motility, such as pyocins, pyoverdine, and the type VI secretion system (3, 54). Interestingly, all these virulence factors can be deployed for competition with other microbial species, like *S. aureus*, which frequently co-infects chronic wounds and the airways of people with cystic fibrosis, alongside *P. aeruginosa* (54, 56–59). Detection of potentially detrimental signals and upregulation of virulence factors to combat them may allow *P. aeruginosa* to move toward the increasingly unfavorable environment and try to eliminate the threat, by killing other microbes or degrading the antibiotics, for example. When monitoring cells experiencing an antibiotic gradient, Oliveira and Wheeler et al. further observed that only a subpopulation of cells moved toward lethal concentrations and died (55). It is possible that this sacrificial strategy may also be employed during chemotaxis toward competitor signals so that only a portion of the population is required to produce virulence factors and potentially die while combating neighboring or invading species.

Additionally, while the mechanisms have not yet been resolved, several other signals have been shown to influence TFP-mediated motility, some of which specifically depend on Pil-Chp components. For example, *P. aeruginosa* was recently demonstrated to move toward an increasing concentration gradient of potassium via TFP, suggesting Pil-Chp may be involved, but was not directly tested (60). Meanwhile, Nolan et al. showed that ChpC is necessary for twitching response to several signals including oligopeptides, mucins, and bovine serum albumin (38). Our study further found that ChpC is a required protein for directed motility toward *S. aureus* PSMs, suggesting that the signals identified by Nolan et al. may also serve as chemoattractant signals sensed by PilJ (38, 42, 51).

## CONTROL OF DIRECTIONAL MOTILITY ON A SURFACE

### Cellular reversals

Like the studies from Howard Berg on swimming bacteria, single-cell tracking of surface-attached twitching cells provides insight into how cells control the direction of motility on a surface. Twitching cells have been shown to bias the direction of movement toward an increasing gradient of attractant for extended periods of time; however, the twitching “runs” up chemoattractant gradients are interrupted by reversal events (Fig. 2A) (3). A reversal occurs when cells perform a directional reversal without a change in the cell body orientation. More specifically, cells will stop moving when they detect a reduced chemical concentration and switch which pole dictates movement, the leading pole, to allow the cell to change course and twitch in a new direction (Fig. 2A). This strategy is consistent with “pessimistic” chemotaxis, in which cells normally perform runs, but the runs are interrupted when cells detect that chemoattractant concentrations are decreasing, at which time cells undergo reversals (Fig. 2A) (3). In contrast, flagellar chemotaxis strategies are considered “optimistic” in that cells stochastically perform reversals until they sense an increasing chemoattractant gradient and then will increase runs and decrease reversals (Fig. 2A) (3). Mathematical modeling predicts the strategy employed by swimming cells produces a larger chemotacic drift, while the strategy used by twitching cells allows for tighter aggregation where the chemoattractant concentration is the highest (61). This model provides useful insight into how these divergent chemotactic strategies may have developed.

Another study utilizing surface-attached cells under flow further uncovered evidence for how twitching reversals may occur. Comparison of twitching cell trajectories of the wild-type and Pil-Chp mutants revealed a role for the response regulators, PilG and PilH, in dictating the reversal frequency. Δ*pilH* lost nearly all ability to reverse, while Δ*pilG* showed approximately twice the number of reversals as the wild type (10). While this study was performed in the absence of a known chemoattractant gradient, these data may suggest that Δ*pilG* cells switch directions so frequently that they have relatively little net change in position and, therefore, could explain why another study observed that Δ*pilG* cells fail to chemotax in the presence of a gradient (3). Meanwhile, wild-type *P. aeruginosa* exhibits nearly double the number of reversals when going away from the chemoattractant gradient as it does when moving toward the increasing gradient (3).

### Step sizes

In our studies on *P. aeruginosa* chemotaxis toward interspecies signals on agarose pads, reversals were not detected at a frequency sufficient to make statistical comparisons of reversal rates between cells traversing up or down chemical gradients. Nonetheless, cells displayed a similar directional bias when faced with an interspecies signal gradient. It was determined that *P. aeruginosa* exhibited increased twitching step sizes, the distance traveled when a singular extension and retraction “step” was taken, when moving toward *S. aureus* versus away (42). More specifically, over time, there was a higher likelihood for cells to take larger twitching steps up the gradient, while the distance traveled per step taken down the gradient was smaller or less frequent (Fig. 2A) (42). It is possible that increased step sizes represent a chemokinetic response, in which the speed or frequency of locomotion is determined by the strength of the stimulus (1), which may be driven by enhanced pili activity at one pole, resulting in directional motility. While the mechanism is unknown, we envision either a larger retraction force, increased speed of extension and retraction, or occupancy of more TFP machines at one pole could produce increased step sizes. Chemotaxis and chemokinesis may both require PilJ but are differentially used under distinct environmental conditions. Nevertheless, it appears that controlled step sizes and reversals both require cells to establish which pole TFP are actively deployed from to move toward or away from a chemoeffector signal. How such events occur in response to chemical signals is still not completely understood.

### Polarity of Pil-Chp proteins

While reversals and steps are clearly important for *P. aeruginosa*-biased twitching motility, there is much to learn about how pilus activity is controlled at each pole and how signals are integrated between poles to change direction. In *M. xanthus*, TFP-mediated directed surface motility also requires reversals, which are mediated by the Frz chemosensory system in this species. The Frz pathway is under the control of a complex polarity module consisting of at least four major proteins. Each member of the *M. xanthus* polarity module exhibits asymmetrical bipolar localization and shifts in distribution at each pole to establish leading and lagging poles (62). A Ras-like GTPase, MglA, serves as the main determinant for the establishment of the leading pole, where it is found in higher concentrations than at the lagging pole (62). The other three module members collectively control the amount of MglA and its GTPase activity at both ends of the cell to aid in pole switching events (63, 62). While homologs of the *M. xanthus* polarity system are not found in *P. aeruginosa*, it is plausible that a similar interplay of Pil-Chp proteins governs changes in polarity for initiation of TFP reversals during twitching motility and chemotaxis.

**Fig 4:**
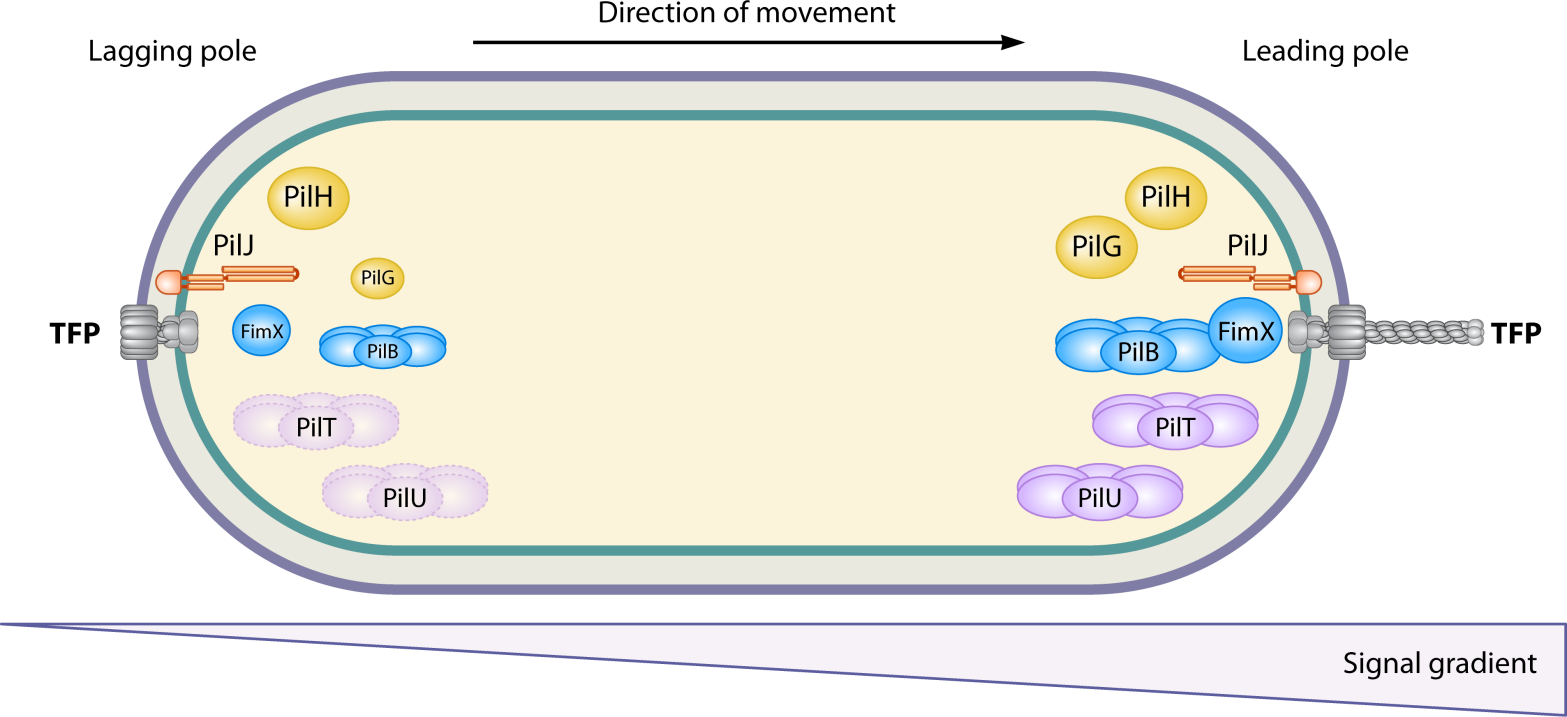
Evidence for a potential TFP polarity module in *P. aeruginosa***.** In twitching cells, there is a leading pole, which faces the same direction the cells are moving in and presumably is the pole with dominant pilus extension and retraction activity (represented by gray pilus filament at the leading pole). The opposite pole is the lagging pole. The leading pole has greater amounts of proteins that control pilus extension (PilB and FimX, azure blue; PilG and PilH, yellow) relative to the concentrations of these proteins at the lagging pole. Meanwhile, the primary retraction ATPase, PilT (light purple), has even distribution at both the leading and lagging pole in surface sensing cells; however, during chemotaxis, PilT localization shifts to be more unipolar at the leading pole. The localization of accessory retraction ATPase, PilU (light purple), is inconsistent in the literature, with one study suggesting PilU bipolarly localizes, while another study shows PilU is unipolar at the leading pole. TFP machinery proteins (gray) like PilQ and PilIO are found equally at both poles along with the MCP, PilJ (orange), which similarly has symmetric, bipolar localization. The polarity differences in cells during chemotaxis compared to surface sensing are still unknown.

Observation of TFP protein localization has started to unveil where the different components are located during motility and reversal events (Table 1). Imaging of fluorescent proteins translationally fused to PilQ and PilO, two members of the core TFP machinery embedded in the membrane, both showed bipolar localization and offered support that TFP complexes simultaneously exist at both poles (Table 1, Fig. 4) (64). The Pil-Chp MCP, PilJ, also localizes to both poles (Table 1, Fig. 4) (65). While the membrane-bound proteins like PilJ and the core machinery are established at both poles continuously, there are several cytoplasmic Pil-Chp proteins, like the response regulators and ATPases, that could more plausibly switch between poles, when needed, such as in response to certain stimuli. This may explain why the Pil-Chp retraction ATPases have different localization patterns across studies (discussed below).

**TABLE 1:** Pil-Chp protein functions and subcellular localization^*a*^

Functional category	Pil-Chp or TFP protein	Protein description	Localization in cell	References
Pil-Chp chemotaxis	PilJ	Methyl-accepting chemotaxis protein (receptor)	Bipolar	(65)
	ChpC	CheW-like linker	Unknown	None
	PilI	CheW-like linker	Unknown	None
	ChpA	Histidine kinase	Unknown	None
	PilH	Response regulator	Cytoplasmic with denser poles	(10, 22)
	PilG	Response regulator	Asymmetric bipolar, denser at leading pole	(10, 22)
	ChpB	Methylesterase	Asymmetric bipolar, denser at leading pole	(23)
	PilK	Methyltransferase	Asymmetric bipolar, denser at lagging pole	(23)
TFP machinery	PilB	Extension ATPase	Asymmetric bipolar, denser at leading pole	(10, 66)
	PilT^*b*^	Retraction ATPase	Bipolar; asymmetric bipolar, denser at leading pole	(10, 67)
	PilU^*b*^	Retraction ATPase	Unipolar (plasmid); bipolar (chromosome)	(10, 66)
	PilO	Alignment complex protein	Bipolar	(64)
	PilQ	Outer membrane porin	Bipolar	(64)

^*a*^ Please note that this is not a comprehensive list of all TFP proteins. Proteins not listed here do not have known subcellular localization.

^*b*^ PilT and PilU each have multiple differing reports regarding their protein localization patterns.

*Pilus extension proteins.* As mentioned briefly above, current data suggest a role for PilG in modulating the localization of the extension ATPase PilB. Both PilB and PilG, along with a c-di-GMP-binding protein, FimX, have been found to localize asymmetrically to the poles, with concentrated localization at the leading pole of cellular movement (Table 1, Fig. 4) (10, 68, 69). Additionally, the loss of either PilB or FimX delocalized the other protein (69), which reflects their reciprocal requirement for proper recruitment at the leading pole. Similarly, loss of PilG or PilG phosphorylation also delocalized PilB and FimX and disrupted TFP motility (10). Interestingly, recent data demonstrate that PilG is also necessary for the localization of the methylesterase ChpB at the leading pole and switches poles during a reversal, suggesting differential methylation of PilJ at the leading and lagging poles may facilitate reversal events (23). For PilH, the majority is localized to the cytosol. Kühn et al. propose that the accumulation of PilH from the cytosol to the pole acts as a brake to inhibit PilG phosphorylation and produce a reversal (10)*.*

*Pilus retraction proteins.* While most available studies demonstrate localization of PilB to the leading pole of movement, the localization of the retraction ATPases is currently less clear. For both PilT and PilU, various studies have reported both unipolar and bipolar localization (Table 1, Fig. 4) (10, 66, 67). It is unclear why the localization patterns of PilU and PilT differ between studies; however, the fusion constructs (multi-copy *in trans* versus single copy *in cis*) and strains (PAO1 versus PA14) vary among these studies. As shown for PilB, PilT has recently been shown to localize to the leading pole of motion and dynamically relocalize during a reversal (65)*.* Thus, differences in localization reported among studies may be a result of these dynamics, which are only captured under certain experimental conditions, time points, or image acquisition parameters.

### Spatial versus temporal sensing

In addition to the contribution of reversals and differences in step sizes to bias twitching motility, it is also necessary to uncover whether twitching cells predominately sense gradients on a temporal or spatial scale. Temporal sensing implies that cells detect significant changes in chemical concentrations over time, that is the cell has the capacity to “remember” the relative chemical concentration it experinced a moment ago. Such temporal sensing requires cells to move through the gradient to sample changes in the chemical landscape over time (Fig. 2B). On the other hand, cells that can sense the gradient spatially would perceive differences in the concentration over a short distance at once and thus can be decoupled from cell movement (Fig. 2B). In *E. coli* and *P. aeruginosa*, swimming cells have been well documented to primarily sense chemoeffector gradients temporally (Fig. 2B) (15, 70–72). *P. aeruginosa* cells tethered to a surface by their flagellar components, yet retaining the ability to rotate their flagellar motor, will rotate their cell body in response to changes in chemoeffector concentration over time (17, 73, 74). Since tethered cells are cemented in space, chemoeffectors flowing through a chamber surrounding the cells will mimic rapid sampling of a gradient in over time (73, 74). In such experiments, the spatially confined, tethered *P. aeruginosa* cells displayed several motor switching events in response to flow-through of L-serine, a known chemoattractant for the flagellar MCP, PctA (73, 74). Such a response supports temporal gradient sensing in flagella-mediated chemotaxis. Based on the speed that flagella-mediated motility occurs, and the distances swimming cells can travel within seconds, cells can sample a wide range of the concentration gradient very quickly (Fig. 2B). Therefore, in relatively short time durations, significant changes in chemoeffector concentrations can be sensed, allowing for more frequent reversals in response.

Meanwhile, TFP-mediated motility has been measured and determined to take place on a much slower time scale, with twitching speeds measuring hundreds to thousands of times slower than swimming cells (Fig. 2B) (3, 75–77). Oliveira et al. highlight one hypothesis to favor spatial sensing in the TFP chemotaxis strategy, in that cells may sense the gradients across the length of their cell bodies (3). In this model, the PilJ MCPs localized at both poles could simultaneously sense chemoeffectors and relay information about the concentration gradient between poles to determine which one is leading. To support that spatial sensing is part of TFP chemotaxis strategy, the localization of the PilT retraction ATPase was monitored as an indicator of pole switching and reversal events in response to a chemical gradient (67). By establishing steep chemical gradients through specialized microfluidics devices, the authors determined that twitching cells were capable of sensing a concentration gradient (reflected by intracellular reversal of PilT localization) independent of changes in cell movement or time (Fig. 4) (67). Steep gradients necessary for spatial sensing may be present in densely packed biofilms due to local consumption of solutes. Additionally, in early studies of twitching chemotaxis by both *Myxococcus* and *Pseudomonas* toward lipids, Kearns et al. proposed that twitching cells may be capable of sensing gradients of lipids due to their low diffusion rate, a property shared by *S. aureus* PSMs (25). Whether spatial sensing is the sole mechanism for deciphering gradients or works jointly with temporal sensing, it is clear that TFP chemotaxis on a surface utilizes a unique strategy apart from flagella-driven directional motility in liquid environments.

## CONCLUDING REMARKS

The earliest studies investigating the Pil-Chp system indicated that this pathway served as a chemosensory system; yet empirical evidence for a role in chemotaxis has emerged primarily in the last decade. Thus, Pil-Chp is a multi-functional manager of distinct signaling responses to surface cues and chemoeffectors sensed by the MCP and surface sensing receptor, PilJ. It is still unknown how many signals constitute the profile of ligands that bind PilJ for both chemoeffectors and surface signals, and the differences in how each signal type interacts with PilJ to initiate signal transduction. However, future work will likely broaden this collection of signals and their PilJ-binding modes of action. In addition, as the field continues to dissect the comparatively complex Pil-Chp chemotaxis and surface sensing system, we will likely gain more insights into the intricacies of how each of the downstream proteins modulate function and polarity of themselves and other Pil-Chp proteins, allowing for further understanding of how twitching cells execute effective movements in response to a variety of different PilJ inputs.

## AFTER EPUB

*[This article was published on 19 September 2025 with errors in the references. The errors were corrected in the current version, posted on 17 October 2025.]*
